# Toxicity of two heavy rare earth elements to freshwater mussels *Dreissena polymorpha*

**DOI:** 10.1007/s11356-024-33633-y

**Published:** 2024-05-18

**Authors:** Houda Hanana, Joëlle Auclair, Patrice Turcotte, Christian Gagnon, François Gagné

**Affiliations:** Environment and Climate Change Canada, 105 McGill, Montréal, Québec H2Y 2E7 Canada

**Keywords:** Mussels, Oxidative stress, DNA damage, Mitochondria activity, Cell division

## Abstract

Rare earth elements (REE) are essential components of many electronic devices that could end-up in solid waste disposal sites and inadvertently released in the environment. The purpose of this study was to examine the toxicity of two heavy REEs, erbium (Er) and lutetium (Lu), in freshwater mussels *Dreissena polymorpha*. Mussels were exposed to 14 days to increasing concentration (10, 50, 250, and 1250 µg/L) of either Er and Lu at 15 °C and analyzed for gene expression in catalase (CAT), superoxide dismutase (SOD), metallothionein (MT), cytochrome c oxidase (CO1), and cyclin D for cell cycle. In addition, lipid peroxidation (LPO), DNA damage (DNAd), and arachidonate cyclooxygenase were also determined. The data revealed that mussels accumulated Er and Lu similarly and both REEs induced changes in mitochondrial COI activity. Er increased cell division, MT, and LPO, while Lu increased DNAd and decreased cell division. Tissue levels of Er were related to changes in MT (*r* = 0.7), LPO (*r* = 0.42), CO1 (*r* = 0.69), and CycD (*r* = 0.31). Lu tissue levels were related to changes in CO1 (*r* = 0.73), CycD (*r* =  − 0.61), CAT (*r* = 0.31), DNAd (*r* = 0.43), and SOD (*r* = 0.34). Although the lethal threshold was similar between Er and Lu, the threshold response for LPO revealed that Er produced toxicity at concentrations 25 times lower than Lu suggesting that Er was more harmful than Lu in mussels. In conclusions, the data supports that the toxicity pattern differed between Er and Lu although they are accumulated in the same fashion.

## Introduction

Rare earth elements (REE) are key ingredients for miscellaneous electronic devices as semi-conductors, plasma screens, cell phones, LED and batteries (Smith Stegen 2015), due to their unique magnetic, luminescent, and catalytic properties. Those superior properties that can provide performance or longevity benefits for electronic parts*,* making them difficult to substitute in some usage (Smith Stegen 2015). As consequence, the extraction of REE in the world was significantly increased in 2021, reaching 280,000 tons by comparison to the 170,000 tons extracted, in 2018 (Duchna and Cieślik 2022). Due to their extensive and growing use around the globe in the last two decades, REEs are now considered as contaminants of emerging concern (MacMillan et al. 2017). Indeed, these elements enter the environment through disposal of consumer and industrial products (e.g., landfills), discharges from mining and mineral processing, and effluents/wastewaters from industrial processes (Gwenzi et al. 2018). Thus, significant levels of anthropogenic REE have been detected in river and lake waters worldwide and reached coastal seawater, groundwater, and tap water (Kulaksız and Bau 2007, 2011; Tepe et al. 2014). Currently, developed countries import the majority of their supplies from China. However, due to the rising demands of REEs and decreasing export from China, new mining is under development around the world to increase domestic supplies (US EPA 2012). Anthropogenic release of REE in the environment could produce adverse effect on aquatic organisms, especially in sessile organisms and filter-feeders such as bivalves.

Recent laboratory studies show bioaccumulation and toxicity of REEs in many species, including aquatic invertebrates as well as in fish and humans (MacMilan 2017) raising environmental health concerns (Pagano et al. 2015a, b). These environmental and occupational health problems commonly result from insufficient environmental regulations and controls in the areas where REEs are mined and processed (Rim et al. 2013). Broadly neglected as xenobiotics until recently, there is a scarcity in the database related to the their effects on human health and the environment (Rim 2016). Historically, REEs have largely been considered an homogenous group of elements of minor risk to environmental or human health because they are lithophilic (i.e., of stony origin), hence largely insoluble and immobile (i.e., not bioavailable) in soils (Šmuc et al. 2012) and are only slightly toxic to mammals (Haley 1979). Most available literature in environmental toxicology are confined mainly to Ce and La, with lesser information available for Gd and Nd, and scanty data available for the other REE, especially for heavy REE (Pagano et al. 2015b) despite their growing uses in a number of advanced technologies (Gravina et al. 2018). It was expected that individual REE displays similar behavior in the ecosystems as well as biological effects due to their “uniform” chemical properties (Blinova et al. 2018). However, recent studies using zebra mussel and rainbow trout indicate that lanthanides displayed different mechanisms of actions. The published data showed that REE-associated effects appear to follow hormetic trends for a number of endpoints (Pagano et al. 2015b). In aquatic organisms, mechanisms of action associated to REEs involve alteration of redox homeostasis (Pagano et al. 2016), modulation of antioxidant activities such as SOD, CAT, and GST (Henriques et al. 2019; Hongyan et al. 2002), alteration of lipid peroxidation (LPO; Figueiredo et al. 2018), and interaction with Ca-dependent biological systems and interfere with cell metabolism (Ippolito et al. 2007; Wang et al. 2011).

In the present study, we investigated the bioavailability and toxicity of two heavy lanthanides (Lu and Er) in freshwater mussels *Dreissena polymorpha*. A number of key toxicological end points were considered such as redox/metal homeostasis, cell proliferation, energy metabolism, inflammation, and DNA damage (DNAd) using a suite of gene expression and biochemical biomarkers. The null hypothesis statement is that the bioavailability and toxicity of two similar heavy REEs, Er and Lu, are undistinguishable.

## Material and methods

### Mussel handling, acclimatation, and exposure to rare earth elements.

Adult zebra mussels, *Dreissena polymorpha* (1.5–2.5 cm shell length), were collected in August 2017 attached to rocks from a reference site in the Saint-Lawrence River near the City of Montréal, Québec, Canada (45° 19′ 50″ N. 73° 58′ 12″ W). Mussels were transported in containers filled with river water to the laboratory. Mussels were allowed to stand 1 month prior to exposures to Er and Lu (as trivalent chloride salts). The background levels of Er and Lu in tissues were below the detection limit of the methodology for these elements using mass spectrometry (< 1 ng/g wet weight). Mussels maintained at 15 °C under constant aeration and were fed with phytoplankton (Phytoplex, Kent Marine, WI) and *Pseudokirchneriella subcapitata* algal preparations every 2 days. This temperature was selected because freshwater mussels are an ideal temperature for long-term maintenance of dreissenids in our hands and is also suitable for salmonids (mussels are sometimes used as fish surrogates). For the exposure experiments, mussels were delicately separated by cutting the byssal treads with a scalpel and immersed in 4-L polyethylene beakers. The mussels (*n* = 20) were treated to LuCl_3_ and ErCl_3_ (10, 50, 250, and 1250 µg/L). Since mass concentrations are usually used for environmental risk assessment, the concentration range corresponds to 0.058–7.5 µM and to 0.057–7.1 µM for Er and Lu respectively) for 14 days at 15 °C. The mussels were not feed during the exposure period. These concentrations were selected based on toxicity range finding using a semi-logarhythmic distribution. The media were renewed every 3 days (semi-static exposure) as follows: 2/3 of the surface water was removed and replaced with fresh Er- or Lu-spiked aquarium water. The control group consisted of mussels exposed to aquarium water (dechlorinated and UV-treated tap water). The exposures were conducted in triplicates (three controls and three vessels for each concentration). Tissue levels in Er and Lu were determined in tissues using ion-coupled plasma mass spectrometry as previously described (Hanana et al. 2018). The mussels were allowed to depurate overnight in clean aquarium prior to mussel tissues collection and microwave-assisted acid digestion in HCl/HNO_3_. The data were expressed as µg of Er or Lu/g wet weight soft tissues. The analysis of other elements was also performed with this methodology encompassing the following elements: sodium (Na), potassium (K), calcium (Ca), magnesium (Mg), manganese (Mn), iron (Fe), cobalt (Co), copper (Cu), silver (Ag), and zinc (Zn).

The persistence of Er and Lu in the exposure media was examined by adding the nominal concentrations of Er and Lu as trichloride salts but with no mussels. The media was changed as with the mussel exposure experiments every 3 days up to day 12, and the levels of Er and Lu were determined in the aquarium water using ICP mass spectrometry as described above. Hence, the reported concentrations in water correspond to the nominal concentration of the REE after 2 days at 15 °C under constant aeration.

High mortality rates (> 70%) were observed after 14 days exposed to 1250 µg/L for both Er and Lu (i.e., were not used for sublethal effects assessments). For the remaining concentrations, mussels were collected (*N* = 10/tank) for biomarker analyses. A total of 30 mussels per concentration of Er and Lu for elemental analysis (*N* = 10 per vessels), gene expression, and biomarker measurements (*N* = 10 each). Biomarkers were performed in the soft tissues and immediately stabilized in RNA Later solution (Thermo Fisher Scientific. Ontario. Canada) after wet weight determinations and stored at − 20 °C. The soft tissues for REE uptake and effect biomarkers were stored at − 85 °C until analysis.

### Physiological biomarkers

The tissues were homogenized using a Polytron tissue grinder in 25 mM Hepes–NaOH, pH 7.4, 100 mM NaCl, 0.1 mM dithiothreitol, and 1 µg/L of aprotinin. The following biomarkers of toxic stress were determined by lipid peroxidation (LPO), DNA strand breaks (DNAd), arachidonate cyclooxygenase (COX), and glutathione *S*-transferases (GST) activities. The homogenate was centrifuged at 15,000 × *g* for 20 min at 2 °C for COX and GST enzyme activities. Total proteins were used to normalize the above endpoints as described previously (Bradford 1976). LPO levels were evaluated using the thiobarbituric acid reactants for malonaldehyde (Wills 1987). Calibration was achieved using tetramethoxypropane solutions and fluorescence readings were taken at 540-nm excitation and 600-nm emission in 96-well dark microplates. Data were expressed as nmoles of TBARS per mg of homogenate proteins. The levels in DNAd were determined using the alkaline precipitation assay (APA) with non-radioactive (fluorescence) detection of DNA strands (Olive 1988; Gagné and Blaise 1995). The measurement of DNA strands remaining in the supernatants was achieved with the Hoechst dye at 350-nm excitation and 450-nm emission in 0.3 M NaCl, 0.2 M Tris base, and 4 mM sodium cholate to minimize interferences from traces of SDS and pH (Bester et al. 1994). Data were expressed as µg DNA × mg^−1^ proteins in the homogenate. GST activity was performed by the method described by Gagné (2014) using 1 mM of 1-chloro-2.4-dichloronitrobenzene (CDNB) as substrate and 1 mM reduced gluthathione in Hepes–NaoH, pH 6.8. The absorbance was measured at 340 nm. and the results were expressed as increased absorbance/minute/mg protein. For the COX activity, the assay measured the formation of H_2_O_2_ from the oxidation of arachidonic acid as previously described (Hanana et al. 2021). Fluorescein was measured at 485 nm for excitation and 520 nm for emission using a microplate reader (Synergy 4, BioTek, USA). The data were expressed as increase in relative fluorescence units (RFU)/ (min × mg proteins).

### Gene expression analysis

Gene expression analysis for catalase (CAT), superoxide dismutase (SOD), metallothionein (MT, cytochrome oxidase (CO1), and cyclin D (CycD) for cell division were determined using qPCR as previously described (Livak and Schmittgen 2001; Hanana et al. 2018). The reactions were determined in duplicate, and the mean from *N* = 10 individuals was calculated. Three housekeeping genes were considered for normalization: elongation factor (EF1α), ribosomal protein *S3*, and *18S*, but only the *EF1α* was selected for the normalization of samples due to its constant expression evaluated by the GenEx software.

### Data analysis

Toxicity data was expressed as effect thresholds as defined: threshold = (no effect concentration x lowest significant effect concentration)^1/2^. The biomarker data were analyzed using a rank-based analysis of variance followed by the Conover-Iman test to find differences from the controls. Possible relationships between tissue levels of Er or Lu and the biomarker data were determined using the Pearson-moment procedure. The biomarker data were also analyzed by principal component analysis to seek out the biomarkers that describe best each element. Significance was set at *p* < 0.05. All the statistical analyses were conducted using SYSTAT (version 13, USA).

## Results

**Fig. 1:**
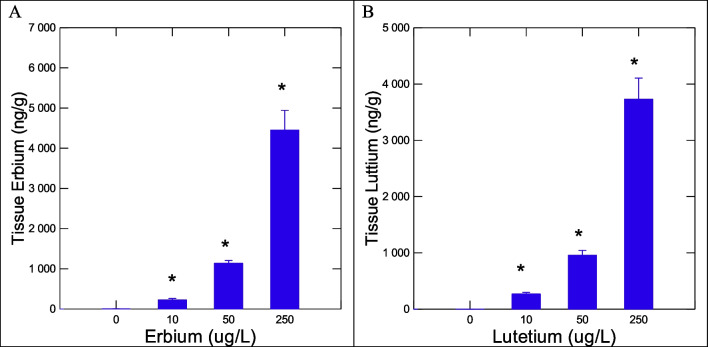
Tissue levels of Er and Lu in freshwater mussels. The levels of Er and Lu were determined in the soft tissues of zebra mussels. The data represent the mean and standard. The star symbol * indicates significance (*α* < 0.05)

In this study, the bioavailability and toxicity of Er and Lu were examined in zebra mussels. These elements are considered heavy REEs with molecular weights of 167 and 175 gmol^−1^ for Er and Lu respectively (Table 1). They are expected to share similar properties with similar ionic radius, redox potential, and electronegativity. Lu has higher calcium-binding potential and slightly higher thiol (glutathione)-binding potential compared to Er. The calcium-binding potential was estimated at 11% and 14% of calcium-binding coefficient to trypsin, a calcium-binding protein, for Er and Lu respectively. For further comparison, iron (Fe) and cobalt (Co) have a calcium-binding potential of 34% of calcium binding to trypsin but with much stronger potential for thiols compared to Ca, Er, and Lu (in increasing order of thiol binding). While Lu shows a stronger affinity to Ca and thiol than Er, they share less affinity to calcium than for Fe, Co, and Ca but shows a stronger affinity to bind thiols than Ca. The persistence of Er and Lu in the exposure media (freshwater) was also investigated (Table 2). After the 1st day of dissolution in aquarium water, the levels of measured concentration of Er represented 70, 80, and 78% of the theoretical added concentrations for 10, 50, and 250 ug/L respectively. This was also the same after 14 days with the exception of 250 ug/L exposure concentration which dropped to 64% of the added theoretical concentration. For Lu, the measured concentrations of Lu represented 70, 77, and 75% of the added theoretical concentration for 10, 50, and 250 ug/L exposure concentration respectively at day 1. After the 14th day, the values were 50, 70, and 94% of the added theoretical values of Lu. The levels of Er and Lu found in the soft tissues of mussels were determined by ICP-MS/MS (Fig. 1). In mussels exposed to Er, tissue levels in Er were significantly increased for all exposure concentrations giving a bioaccumulation factor of 18. For Lu, the same was observed for all exposure concentrations with a bioaccumulation factor of 15. This suggests that these two heavy REEs were equally bioavailable to freshwater mussels. The levels of essential elements were also determined in mussels exposed to either Er and Lu (Table 3). In mussels exposed to Er, Na and Ca were significantly decreased and increased respectively at the highest exposure concentration. In mussels exposed to Lu, the levels of Na, K, and Co were significantly reduced at the highest exposure concentration of 250 ug/L. Correlation analysis revealed that Er was significantly correlated with soft tissue weight index (soft tissues weight/mussel weight) (*r* = 0.42) and Na (*r* =  − 0.31). Lu tissue levels were significantly correlated with soft tissues weight index (*r* =  − 0.47) and Mg (*r* = 0.26).

**Table 1:** Physico-chemical characteristics of Er and Lu

Element	MW	Ionic radius	Electronegativity	Redox	Calcium binding potential^1^	Thiol binding^2^
Er	167	88	1.24	− 2.32	1487	7.32
Lu	175	84.8	1.27	− 2.3	1834	7.42
Ca	40	106	1	− 2.84	13,201	6.89
Fe	55	82(+ 2)/67(+ 3)	1.83	− 0.04	4520	7.87
Co	58	82(+ 2)/64(+ 3)	1.88	− 0.28	4456	7.94

^1^The calcium binding potential was estimated by the binding constants with trypsin, a calcium binding protein (Epstein et al. 1974). A multiple linear regression model was found between trypsin-binding constants and atomic weight and ionic radius: binding constant (mol^−1^) = 15,276 − (11,723 × radius in nm) + (20.8 × MW); *r* = 0. − 91 *p* = 0.002

^2^From Garg et al. 2003 with the linear regression with ionic radius: Log K (GSH) = 9.544 − (0.025 × ionic radius) (*N* = 12 elements, *r* = 0.82)

**Table 2:** Measured concentration of Er and Lu in aquarium water over time

Added element	Measured (µg/L) day 1	Measured (µg/L) day 14
Er		
10 µg/L	7 (70%)^1^	7 (70%)
50 µg/L	40 (80%)	30 (75%)
250 µg/L	195 (78%)	150 (77%)
Lu		
10 µg/L Lu	7.2 (72%)	3.5 (50%)
50 µg/L Lu	41 (82%)	29 (70%)
250 µg/L	161 (64%)	151 (94%)
Controls	Er: 0.0006; Lu: 0.0008	Er: 0.001; Lu: 0.0015

^1^% remaining at day 15 relative to the theoretical added concentration

**Table 3:** Influence of Er and Lu on essential elements in tissues

Essential elements	Erbium				Lutetium			
	0	10	50	250	0	10	50	250
Na (μg/g)	374(7)	370(13)	352(15)	**298(11)**	374(9)	362(12)	339(13)	**348(10)**
K (μg/g)	105(8)	95(5)	105(6)	91(5)	105(3)	105(7)	110(9)	**92(4)**
Ca (μg/g)	251(3)	250(14)	259(9)	**293(6)**	251(12)	283(23)	268(22)	272(21)
Mg (μg/g)	67(3)	64(5)	65(5)	65(3)	67(3)	70(4)	69(4)	70(3)
Fe (× 103) (ng/g)	10.4(0.6)	10.2(1.2)	10.1(1.1)	9.9(0.8)	10.2(0.4)	10.1(0.7)	8.4(0.4)	10.3(0.7)
Co (ng/g)	61(5)	52(6)	52(6)	61(5)	59(4)	53(5)	**41(4)**	**46(3)**
Cu (ng/g)	510(26)	498(42)	538(29)	511(30)	510(26)	530(24)	450(26)	512(31)
Zn (× 103) (ng/g)	5.2(0.3)	5(0.5)	4.9(0.3)	4.8(0.4)	5.2(0.3)	4.9(0.3)	4.6(0.4)	4.9(0.2)

The data represent the mean (ug/g wet weight) with the standard error in parentheses. **Bold** values indicate significance from the controls

**Fig. 2:**
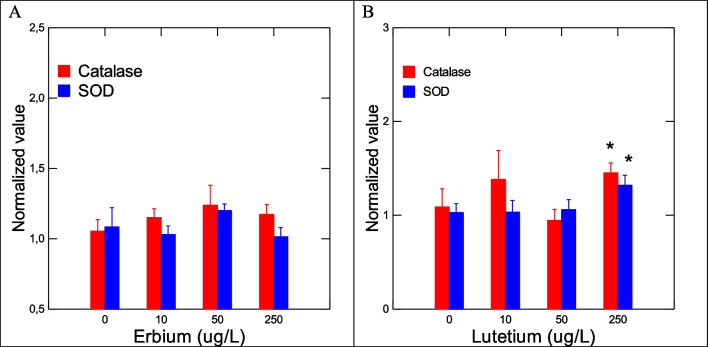
Oxidative stress responses of Er and Lu in freshwater mussels. Oxidative stress was determined by following gene expression of catalase and SOD. The data represent the mean and standard. The star symbol * indicates significance (*α* < 0.05)

**Fig. 3:**
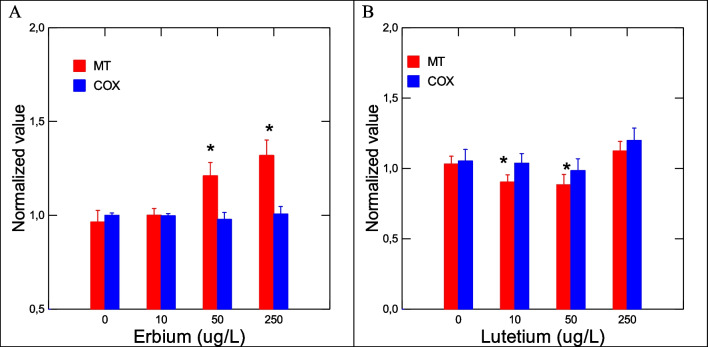
Metal homeostasis and inflammation responses in freshwater mussels. Metal homeostasis and inflammation were determined by MT levels and arachidonate cyclooxygenase activity. The data represent the mean and standard. The star symbol * indicates significance (*α* < 0.05)

**Fig. 4:**
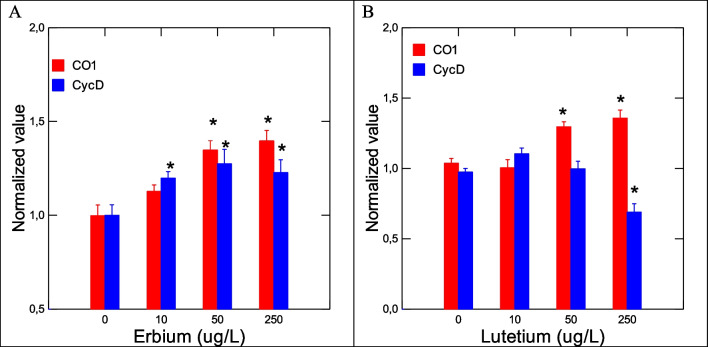
Change in gene expression for mitochondria activity and cell growth. Mitochondria activity and cell growth were examined by CO1 and CycD gene expression. The data represent the mean and standard. The star symbol * indicates significance (*α* < 0.05)

**Fig. 5:**
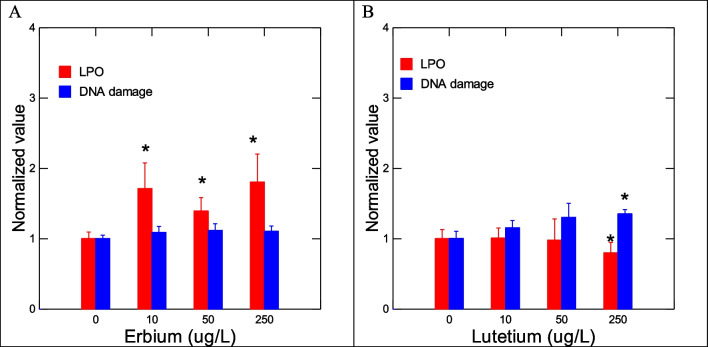
Oxidative damage and genotoxicity in mussels exposed to Er and Lu. Oxidative damage and genotoxicity were determined by following changes in LPO and DNA stand breaks. The data represent the mean and standard. The star symbol * indicates significance (*α* < 0.05)

The toxic effects of Er and Lu in zebra mussels were determined by a suite of gene expression assays (CAT, SOD, MT, CO1, and CycD) and biochemical assays (COX, APA, LPO) destined to target oxidative stress (CAT, SOD, MT, COX), DNAd, mitotic cell divsion (CycD), and energy metabolism/respiration (CO1). Genes involved in oxidative stress were examined in mussels exposed to each of the 2 REEs (Fig. 2). No significant changes in SOD and CAT gene expression were observed in mussels exposed to Er. In mussels exposed to Lu, gene expression in SOD and CAT was increased at the highest exposure concentration. Correlation analysis revealed that SOD was significantly correlated with Lu in tissues (*r* = 0.32). CAT gene expression was correlated with Lu in tissues (*r* = 0.49), Na (*r* =  − 0.3), Mg (*r* = 0.34), and Fe (*r* = 0.34). Furthermore, gene expression in MT, involved in redox and divalent metal homeostasis, and COX activity were also determined (Fig. 3). In mussels exposed to Er, MT mRNA levels were significantly increased at 50 and 250 ug/L exposure concentration with no significant changes in COX activity. However, correlation analysis revealed only that COX activity was related to Lu in tissues (*r* = 0.32). Mitochondria (respiration and energy production) and cell division/growth (CycD)) were determined in mussels exposed to either Er to Lu. In mussels exposed to Er, mitochondria activity (CO1 gene expression) was significantly increased at 50 and 250 µg/L Er while cell division was stimulated at all Er concentrations. In mussels exposed to Lu, CO1 gene expression was increased at 50 µg/L and 250 µg/L while cell division was inhibited at 250 µg/L (Fig.  4). Correlation analysis revealed that CO1 gene expression was correlated with Mg in tissues (0.29), Er in tissues (*r* = 0.43), Lu in tissues (*r* = 0.36), CAT (*r* = 0.53), and SOD (*r* = 0.29). CycD gene expression was significantly correlated with Lu in tissues (*r* =  − 0.58), Er in tissues (*r* = 0.26), and COX activity (*r* = 0.31). Tissue damage was determined by following changes in LPO and DNAd (Fig. 5). In mussels exposed to Er, LPO was elevated at all exposure concentrations but no DNAd. In mussels exposed to Lu, LPO and DNAd were significantly decreased and increased respectively at the highest exposure concentration. Correlation analysis revealed that LPO was correlated with Er in tissues (*r* = 0.4) and MT gene expression (*r* = 0.59). DNAd was significantly correlated with CO1 (*r* = 0.26) and Er (*r* = 0.30).

**Fig. 6:**
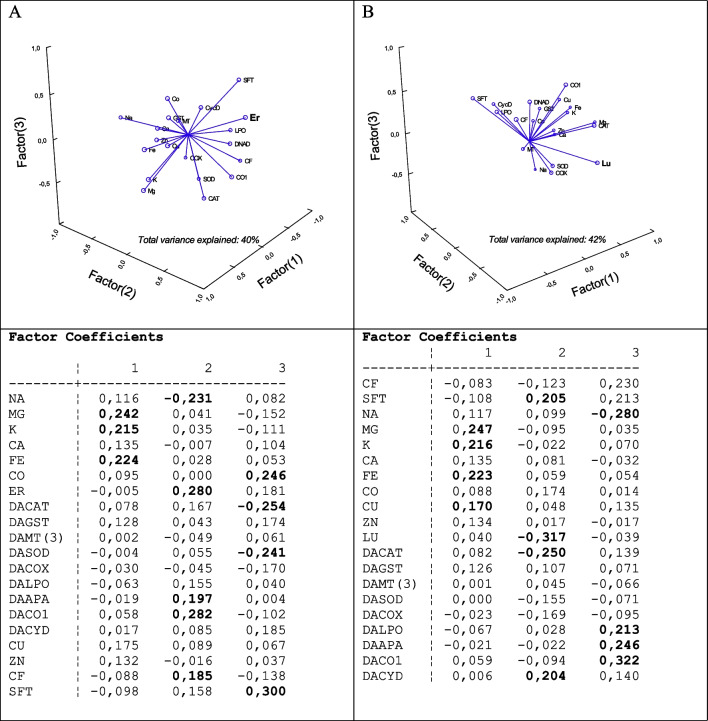
Principal component analysis of biomarker responses in mussels. Principal components were determined to find the most important biomarkers with the highest factorial weights and those closely associated to tissue levels in Er and Lu.

In the attempt to gain a global view on the toxicity of Er and Lu, a principal component analysis was performed (Fig.  6). In mussels exposed to Er, 40% of the total variance was explained by the following biomarkers in decreasing order of factorial weights: Er in tissues > CO1 > CAT > Co > SOD > Mg. In mussels exposed to Lu, the total variance was explained at 42% by the following biomarkers in decreasing order of factorial weights: Lu in tissues > CO1 > Na > CAT > Mg > DNAd. Hence, the tissue loadings in the exposure of Er and Lu were the most important endpoints with the following biomarkers affected by both elements: Co1, CAT, and Mg. Er involved more strongly SOD and Co while Lu involved Na in tissues and DNAd.

## Discussion

The toxicity of Er and Lu was examined with a suite of biomarkers in freshwater mussels where some aspects of the responses differed between these two heavy REES. For example, both Er or Lu produced increased CO1 gene expression involved in energy metabolism and respiration in mitochondria but only Lu reduced CycD expression involved in cell division and Co levels in tissue (Bragina et al 2015). The essential element Co is found in vitamin B12 and act as cofactor involved in methionine (an essential amino-acid initiating protein synthesis) and tetrahydrofolate for DNA synthesis (Bandarian and Matthews 2013). While mitochondria spend more energy (ATP) as suggested by increased CO1 gene expression by both Er and Lu, this energy was less used for protein and DNA synthesis in mussels exposed to Lu (Sokolova et al. 2012). This was further supported by decreased CycD gene expression involved in mitotic cell division. Exposure of Er leads to increased Ca, MT, and LPO levels and decreased Na in tissues. In another study, gadolinium was shown to decreased the rise in Na + in muscle fibers during contraction (Yeung et al. 2003). Gadolinium, a REE closer in mass (MW 157.3 gmol^−1^) to Er than to Lu but with higher ionic radius (105 pm), was also shown to inhibit Na, Ca-dependent conductance in proximal tubular cells from the Rana temporaria frogs (Robson and Hunter 2000). Based on the data in Table 1, the calcium-binding potential of Gd (1731 mol^−1^) was higher than Er (1478 mol^−1^) but lower than Lu (1834 mol^−1^). The effects of gadolinium impacted more calcium conductance than Na suggesting that Na/Ca exchanges were dampened, which could account for loss of Na and increase Ca in soft tissues. Indeed, the inhibition of Na/Ca exchanger was consistent with the observed increase in intracellular Ca and decrease intracellular Na. Hence, it is suggested that Er shares similar properties to gadolinium. Er had potent effects on mitochondria activity (CO1 gene expression) and cell division/growth (CycD). The increase in LPO by all concentrations of Er suggests oxidative damage in cells, and MT gene expression was induced only at the highest concentration. In a previous study, Er did not induce MT gene expression as with the present study but was toxic to rainbow trout juveniles with a LC50 of 8 mg/L (Dubé et al. 2019). The mortality threshold of Er was estimated at 0.56 mg/L in the present study. It was shown that Er-coated cerium nanoparticles enhanced the rapid catalysis of reactive oxygen species (Li et al. 2020) but was not immediately compensated by induction in CAT or SOD gene expression, which resulted in oxidative damage (LPO). MT was recognized not only as heavy metal scavenger but a reactive oxygen scavenger during inflammation as well (Kobayashi et al. 2005; Nielsen et al. 2007). This is in agreement with the observed correlations between MT gene expression and LPO (*r* = 0.59) in the present study. This was also found with Er oxide nanoparticles, i.e., induced oxidative stress leading to cell cycle arrests and apoptosis (Safwat et al. 2022).

In the case of mussels exposed to Lu, decreased Na, K, and Co tissue levels were observed. It was found that terbium inhibited Na pump in kidney membranes, and Ca had minimal effects on rubidium (K +) uptake (Reifenberger et al. 2007). DNAd was not associated to LPO suggesting that damage was not mediated by oxidative stress. This was supported statistically where an analysis of covariance of DNAd using LPO as the covariate revealed a significant effect of Lu exposure concentration only. In a previous study with rainbow trout juvenile exposed to Lu (Hanana et al. 2021), they found that Lu was toxic at 1.9 mg/L and did not influence SOD, CAT, and GST, as observed here, supporting the hypothesis that the genotoxicity of Lu did not follow oxidative stress. It was shown that the hydration of rare earths differed between them (Rudolph and Irmer 2019). The oxygen distance with the lanthanides revealed that Lu-O was the shortest and Er-O distance was significantly higher. This suggests that Er-O could be broken more easily releasing Er + 3 for oxidation processes. In contrast, the closer distance of Lu-O bond could render it less prone to oxidation processes. This is consistent with lower calcium binding constant of Er compared to Lu towards trypsin (Epstein et al. 1974). Current evidence shows that REEs have DNA binding/interaction properties (Vellampatti et al. 2019). In Nd or Er-doped DNA films, the UV-photovoltage responses were increase fivefold for Nd^3+^, a light REE, and tenfold with the heavy REE Er^3+^. This suggests that heavy REEs interact more closely with UV-produced electron transfers on DNA films. In another study, Er3 + and tryptophan complex were shown to bind herring sperm DNA in vitro (Zhao et al. 2010). The complex was shown to bind trough intercalation in the DNA grooves. Hence, the capacity of heavy REEs to interact with DNA, the shorter distance of the Lu-O (preventing less charge transfers), and increased gene expression in CAT and SOD to prevent the formation of oxygen radicals in the case of Lu could form the basis of increased DNAd in mussels. Er was shown to decrease SOD activity in Daphnia magna exposed for 21 days (Galdiero et al. 2019) suggesting increased susceptibility to oxidative stress. This was supported in mussels exposed to Er had significantly higher LPO levels at all exposure concentration but with no genotoxicity. Based on the observed responses, the toxicity threshold of Er and Lu was at 560 µg/L based on mortality (shell opening) based on the following: threshold = (lowest observed effects × no effect concentration)^1/2^. The same was observed with the lethal and sublethal toxicity with the invertebrate *Hydra attenuata* with an LC50 and EC50 of 340 and 100 µg/L respectively (Blaise et al. 2018). The toxicity of Er was somewhat less than Lu but in the same order of magnitude in rainbow trout juveniles with an LC50 of 8 and 4 mg/L respectively (Dubé et al. 2019). Based on the observed responses, sublethal effects are occurring at concentration 75 times below the lethal toxicity threshold for Er and Lu respectively. This corresponds to tissue levels of 228 (95% CI 187–270) and 268 (95% CI: 226–311) ng/g in soft tissues.

In conclusion, Er and Lu were readily absorbed in tissues of freshwater mussels with bioaccumulation factors of 15 and 18 respectively. The presence of these elements in tissues produced both common and specific effects. Both elements were able to induce similar changes in CO1 transcript levels suggesting increased metabolic activity and energy expense (ATP production). Specific effects of Er exposure were increased MT, CycD transcripts, and LPO levels while Lu specifically increased CAT, SOD transcripts, DNAd, and Ca levels in tissues. However, Er decreased Na and K levels in tissues. Lu was also able to decrease CycD gene expression involved in mitotic cell division, Na, K, and Co levels in tissues. This suggests that both elements were able to increase energy expenses, while Er and Lu increased and decreased cell division (CycD) respectively. On the one hand, exposure to Er did activate reactive oxygen species deactivation (SOD, CAT) and lead to oxidative stress as determined by LPO. On the other hand, Lu activated the pathway involved in the inactivation of reactive oxygen species protecting against further damage such as LPO but nevertheless produced DNAd, which was seemingly independent of reactive oxygen radicals. This suggests that heavy REEs could have different toxicities in freshwater mussels.

## Data Availability

Data availability is available upon request.
